# Once-Weekly Somapacitan vs Daily GH in Children With GH Deficiency: Results From a Randomized Phase 2 Trial

**DOI:** 10.1210/clinem/dgz310

**Published:** 2020-01-09

**Authors:** Lars Sävendahl, Tadej Battelino, Meryl Brod, Michael Højby Rasmussen, Reiko Horikawa, Rasmus Vestergaard Juul, Paul Saenger, REAL 3 study group, Dieter Furthner, Bettina Piringer, Lorenz Auer-Hackenberg, Klaus Schmitt, Marlene Reitmayr, Marcello Delano Bronstein, Francisco Samuel Magalhães Lima, Martin Wabitsch, Carsten Posovszky, Volker Böttcher, Alexander Mann, Eli Hershkovitz, Alon Haim, Neta Lowenthal, Orit Hamiel, Sharon Sheinvald Levin, Kineret Mazor-Aronovitch, Michal Ben-Ami, Yael Levy Shraga, Dalit Modan, Noah Gruber, Moshe Phillip, Yael Lebenthal, Ariel Tenenbaum, Alon Eliakim, Nitzan Dror, Ruby Haviv, Nehama Zuckerman-Levin, Naim Shehadeh, Liav Givon, Ameer Elemy, Miriam Marji, Vardit Gepstein, V P Praveen, P Aswin, Nithiya Abraham, Rajesh Khadgawat, Yashdeep Gupta, Vaman Khadilkar, Anuradha Khadilkar, Sagar Lad, Reiko Horikawa, Yasuhiro Naiki, Yasuko Ogiwara, Yuta Chiba, Yusuke Fujisawa, Yumiko Terada, Tomoko Yoshida, Kenichi Kinjo, Atsushi Tsukamura, Shinobu Ida, Yuri Etani, Yasuko Shoji, Masanobu Kawai, Hisakazu Nakajima, Jun Mori, Shota Fukuhara, Keiichi Shigehara, Hidechika Morimoto, Yusuke Tsuma, Yasuhiro Kawabe, Takeshi Ota, Kenichi Kashimada, Ryuichi Nakagawa, Atsumi Tsuji, Risa Nomura, Kei Takasawa, Takeru Yamauchi, Kanako Ishii, Naoko Toda, Kazuhiro Ohkubo, Tohru Yorifuji, Yuki Hosokawa, Rie Kawakita, Yukiko Hashimoto, Azumi Sakakibara, Shinji Higuchi, Shun Soneda, Kenichiro Ogushi, Shuichi Yatsuga, Yasutoshi Koga, Takako Matsumoto, Miyuki Kitamura, Lars Sävendahl, Ricard Nergårdh, Tadej Battelino, Mojca Zerjav Tansek, Serap Turan, Abdullah Bereket, Zeynep Atay, Azad Akbarzade, Olena Bolshova, Mykola Tronko, Olga Vyshnevskaya, Natalia Sprynchuk, Iryna Lukashuk, Natalia Muz, Tatyana Marchenko, Nataliya Chorna, Marіana Konovalova, Liliya Zelinska, Lawrence Silverman, Barbara Cerame, Sunita Cheruvu, Daisy Chin, Laurie Ebner-Lyon, Marie Fox, Marianna Nicolette-Gentile, Kristin Sabanosh, Harold Starkman, Ian Marshall, Mariam Gangat, Sadana Balachandar, Philippe Backeljauw, Andrew Dauber, Leah Tyzinski, Paul H Saenger, Luis Zamora Siliezar, Jacqueline P Velasco, Judith L Ross, Martha Bardsley, Karen Kowal, Gad B Kletter, Britney G Frazier, Kathryn Garrison

**Affiliations:** 1 Department of Women’s and Children’s Health, Karolinska Institute and Pediatric Endocrinology Unit, Karolinska University Hospital, Stockholm, Sweden; 2 UMC–University Children’s Hospital, and Faculty of Medicine, University of Ljubljana, Ljubljana, Slovenia; 3 The Brod Group, Mill Valley, California, US; 4 Novo Nordisk, Global Development, Søborg, Denmark; 5 National Center for Child Health and Development, Tokyo, Japan; 6 University Hospital, Mineola, New York, US

**Keywords:** growth hormone, growth hormone deficiency, growth hormone replacement therapy, long-acting growth hormone, somapacitan, treatment burden

## Abstract

**Context:**

Daily growth hormone (GH) injections can be burdensome for patients and carers. Somapacitan is a long-acting, reversible albumin-binding GH derivative in development for once-weekly administration in patients with growth hormone deficiency (GHD).

**Objective:**

The objective of this study is to evaluate the efficacy, safety, and tolerability of once-weekly somapacitan vs once-daily GH.

**Design:**

REAL 3 is a multicenter, randomized, controlled, double-blind (somapacitan doses), phase 2 study with a 26-week main and 26-week extension phase (NCT02616562).

**Setting:**

This study took place at 29 sites in 11 countries.

**Patients:**

Fifty-nine GH treatment-naive prepubertal children with GHD were randomly assigned; 58 completed the trial.

**Interventions:**

Interventions comprised 3 somapacitan doses (0.04 [n = 16], 0.08 [n = 15], or 0.16 mg/kg/wk [n = 14]) and daily GH (0.034 mg/kg/d [n = 14]), administered subcutaneously.

**Main Outcome Measures:**

The primary end point was height velocity (HV) at week 26. Secondary efficacy end points included HV SD score (SDS) and insulin-like growth factor-I (IGF-I) SDS.

**Results:**

At week 26, mean (SD) annualized HV for the somapacitan groups was 8.0 (2.0), 10.9 (1.9), and 12.9 (3.5) cm/year, respectively, vs 11.4 (3.3) cm/year for daily GH; estimated treatment difference (somapacitan 0.16 mg/kg/week—daily GH): 1.7 [95% CI –0.2 to 3.6] cm/year. HV was sustained at week 52, and significantly greater with somapacitan 0.16 mg/kg/week vs daily GH. Mean (SD) change from baseline in HV SDS at week 52 was 4.72 (2.79), 6.14 (3.36), and 8.60 (3.15) for the somapacitan groups, respectively, vs 7.41 (4.08) for daily GH. Model-derived mean (SD) IGF-I SDS for the somapacitan groups was −1.62 (0.86), −1.09 (0.78), and 0.31 (1.06), respectively, vs −0.40 (1.50) observed for daily GH. Safety and tolerability were consistent with the profile of daily GH.

**Conclusions:**

In children with GHD, once-weekly somapacitan 0.16 mg/kg/week provided the closest efficacy match with similar safety and tolerability to daily GH after 26 and 52 weeks of treatment. A short visual summary of our work is available (1).

Growth hormone deficiency (GHD) in children is characterized primarily by a diminished height velocity (HV), and/or a height below the normal range, and/or below the range expected based on the parents’ height. When given replacement therapy with recombinant human GH (hGH), normal growth can be restored in most cases (2). However, the short half-life of GH necessitates daily subcutaneous injections, which can be burdensome and lead to reduced adherence (3, 4). For example, in 1 study, approximately 25% of children on daily GH treatment missed more than 2 injections per week (5). Reduced adherence, in turn, negatively affects growth (5, 6).

Somapacitan, a once-weekly treatment for GHD in children and adults, is being developed with the aim of reducing injection frequency (7). Once-weekly treatment with somapacitan is expected to have at least the same efficacy and safety profile as daily GH, as well as reducing treatment burden and potentially improving adherence.

Somapacitan is a long-acting human GH derivative to which a small noncovalent albumin-binding moiety is attached. This facilitates reversible binding to endogenous albumin, delaying the elimination of somapacitan, prolonging half-life, and thereby extending duration of action. Similar techniques of conjugating a peptide drug to a small-albumin binding moiety have been used successfully to enhance the half-life of insulin detemir (8) and the glucagon-like peptide-1 molecules liraglutide (9) and once-weekly semaglutide (10). Somapacitan has been shown in previous short-term trials to be well tolerated in healthy adults (11) and in adults and children with GHD (7, 12, 13). A modeling analysis based on observed data from somapacitan phase 1 trials demonstrated that weekly dosing of somapacitan provides elevated insulin-like growth factor-I (IGF-I) levels throughout the week, despite little or no accumulation of somapacitan, both in adults and children with GHD (14).

The objective of this phase 2 trial (REAL 3; NCT02616562) was to investigate the efficacy, safety, and tolerability of 3 different once-weekly subcutaneous doses of somapacitan compared with daily administration of GH (Norditropin; Novo Nordisk A/S) in prepubertal children with GHD.

## Materials and Methods

### Study design and treatments

**Figure 1.**
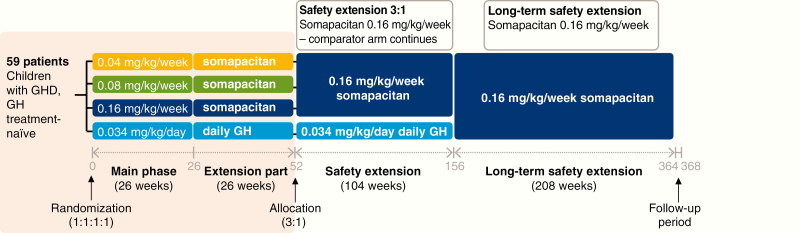
Design of the REAL 3 trial and extensions. The pink shaded box indicates the phases reported in this paper. Time axis is not to scale. GH indicates growth hormone; GHD, growth hormone deficiency.

REAL 3 was a randomized, multinational, active-controlled, open-label, dose-finding, 4-arm parallel group trial in GH treatment-naive, prepubertal children. The trial investigated the safety and efficacy of once-weekly somapacitan treatment at 3 doses (0.04, 0.08, or 0.16 mg/kg/week) vs a control arm of daily GH (0.034 mg/kg/day) (Fig. 1). The doses of somapacitan were selected based on the results of a dose-escalation trial (7), which indicated that the IGF-I response to doses of 0.04, 0.08, and 0.16 mg/kg was comparable to the IGF-I response to daily GH, with no new safety concerns. The daily GH dose of 0.034 mg/kg/day was chosen based on the maximum dose according to the product label (0.034 or 0.035 mg/kg/day in the participating countries). The trial was double-blind with regard to somapacitan dose, and the clinical assessments were conducted by assessors blinded to somapacitan dosing.

The main trial period was 26 weeks, followed by a 26-week extension. This is being followed by an additional extension of 104 weeks comparing somapacitan 0.16 mg/kg/week with daily GH 0.034 mg/kg/day, and a further 208-week, open-label safety extension using somapacitan 0.16 mg/kg/week only (these phases are ongoing and not reported here). The REAL 3 trial was conducted at 29 sites in 11 countries (Austria, Brazil, Germany, India, Israel, Japan, Slovenia, Sweden, Turkey, Ukraine, and the United States) between March 2016 and August 2018.

The 0.04, 0.08, and 0.16 mg/kg/week doses of somapacitan were provided as 5 mg/1.5 mL, 10 mg/1.5 mL, and 15 mg/1.5 mL, respectively, in prefilled pen-injectors of the FlexPro family (Novo Nordisk A/S). Daily GH was provided using Norditropin FlexPro 10 mg/1.5 mL.

Body weight was measured at all visits and used for dosing calculation. Patients were seen every 13 weeks for efficacy measurements, adverse events (AEs), and safety laboratory measurements. The protocol was approved by the local and national ethics committees, as appropriate, and conducted in accordance with the International Conference on Harmonisation Guidelines for Good Clinical Practice and the Declaration of Helsinki. Informed consent was obtained in writing from the parents (and/or the child’s legally acceptable representative), and child assent was obtained as age- appropriate.

### Random assignment and masking

The children were randomly assigned by investigators at the trial sites using a trial-specific, web-based, interactive response system. The randomization was stratified by region (Japan and rest of the world), as well as by age (< 6 and > 6 years), and by sex within the rest-of-the-world region. The pen-injectors used for the 3 doses of somapacitan were visually identical, and assessors performing the height measurements were blinded to treatment allocation.

### Patients

A total of 60 patients were planned for enrollment. Eligible patients were prepubertal children with a confirmed diagnosis of GHD within 12 months before screening, as determined by 2 different unprimed GH stimulation tests (defined as a peak GH level of ≤ 7.0 ng/mL with no prior exposure to GH therapy and/or IGF-I treatment). For children with 3 or more pituitary hormone deficiencies, only 1 GH stimulation test was required. In Japan, the requirement was for confirmed diagnosis of GHD within 12 months before screening was determined by 1 GH stimulation test for patients with intracranial organic disease or symptomatic hypoglycemia, and 2 different GH stimulation tests for other patients, with a cutoff peak GH level of 6 ng/mL or less by assay using a recombinant GH standard.

Other key inclusion criteria were: for boys—Tanner stage 1 for pubic hair and testis volume less than 4 mL, age 2 years and 26 weeks or older and 10.0 years or younger at screening; for girls—Tanner stage 1 for breast development (no palpable glandular breast tissue) and pubic hair, age 2 years and 26 weeks or older and 9.0 years or younger at screening (15); for all children—height of at least 2.0 SD below (corresponds to below third percentile) the mean height for chronological age (CA) and sex according to the Centers for Disease Control and Prevention for ages 2 to 20 years: girls/boys stature-for-age and weight-for-age percentiles at screening (16) and annualized HV at screening below the 25th percentile for CA and sex or below –0.7 SD score for CA and sex, according to the standards of Prader (17), calculated over a minimum time span of 6 months and maximum 18 months before screening.

Children with any clinically significant abnormality likely to affect growth or the ability to evaluate growth with standing measurements were not eligible for the study. Abnormalities of concern included chromosomal aneuploidy and significant gene mutations causing medical “syndromes” with short stature (including but not limited to Turner syndrome, Laron syndrome, Noonan syndrome, or absence of GH receptors); congenital abnormalities (causing skeletal abnormalities), including but not limited to Russell-Silver syndrome and skeletal dysplasias; and significant spinal abnormalities, including but not limited to scoliosis, kyphosis, and spina bifida variants. Other exclusion criteria were children born small for gestational age (birth weight and/or birth length < –2 SD for gestational age); concomitant administration of other treatments that may have an effect on growth, including but not limited to methylphenidate for treatment of attention deficit hyperactivity disorder; and prior history or presence of malignancy and/or intracranial tumor.

Treatment adherence during the trial was monitored by electronic diaries, in which patients were instructed to record the date, time, and dose of injections of the trial drug as well as any missed doses.

### Objectives and end points

#### Primary objective.

The primary objective was to evaluate the efficacy of 3 different doses of once-weekly somapacitan in GH treatment-naive prepubertal children compared to daily administration of GH after 26 weeks of treatment. This was assessed by HV (cm/year), measured as standing height with a stadiometer, at baseline, and at week 26.

#### Other efficacy end points.

Supportive secondary efficacy end points included changes from baseline to end of week 26 and week 52 in height SD score (SDS), HV SDS, change from baseline in HV SDS, IGF-I SDS, and insulin-like binding protein 3 (IGFBP-3) SDS. The HV SDS at week 52 was reanalyzed as a post hoc sensitivity analysis to exclude 4 visits of 1 patient who discontinued prematurely.

IGF-I and IGFBP-3 analyses were performed by a central laboratory using commercially available assay kits (Immunodiagnostic Systems Immunoassay) (18, 19). Samples collected at weeks 4, 13, and 39 were trough levels (day 7 after dosing), and samples collected at weeks 26 and 52 were peak levels (days 1-4 after dosing) for somapacitan. This was planned to provide information on peak-to-trough fluctuations and to derive the average using population pharmacokinetic/pharmacodynamic (PK/PD) modeling. IGF-I SDS was calculated according to the modified least mean squares model with data from 15 014 individuals, as published by Bidlingmaier (18).

#### Population pharmacokinetic/pharmacodynamic modeling.

The modeling was performed to derive average IGF-I levels over the dosing interval following somapacitan treatment. The population PK/PD modeling was based on somapacitan and IGF-I concentrations and the time since last dose for blood samples, using a previously developed model for somapacitan from trials with full PK/PD profiles. This PK/PD model was a 1-compartment PK model with dual first- and zero-order absorption through a transit compartment, and with saturable elimination with an indirect response PD model for somapacitan effect on IGF-I (14). Average IGF-I levels were derived from the predicted area under the curve in a dosing interval for each individual treated with somapacitan.

IGF-I SDS profiles for somapacitan were derived by population PK/PD modeling from the REAL 3 trial. Daily GH IGF-I SDS profiles are shown for reference and were derived by population PK/PD modeling of phase 1 data (daily GH model not published).

Population PK/PD modeling was performed in NONMEM version 7.3 (ICON Development Solutions), PsN version 4.6.0 (20), and R version 3.2.3 (21).

#### Patient-reported outcomes.

The Growth Hormone Deficiency—Child Impact Measure (GHD-CIM) was developed according to US Food and Drug Administration guidance (22) to assess the impact of GHD on physical functioning and social and emotional well-being of children with GHD. The GHD-CIM observer-report (GHD-CIM ObsRO) was developed for parents and guardians of children age 4 to 12 years (inclusive); its development and validation will be reported separately. This manuscript reports the findings of a preplanned analysis performed on data obtained from parents and guardians of all the children in the trial (ages 2-10 years) using the GHD-CIM ObsRO. The results were interpreted on the basis of minimal important difference (MID) in rating. Patient and clinician ratings of severity of disease suggest an MID of 7 for the emotional well-being, 5 for the physical health, and 5 for the social well-being domains, and 5 points for the total score of the GHD-CIM ObsRO.

#### Supportive secondary safety end points.

These included incidence of AEs, including injection-site reactions; occurrence of antisomapacitan and anti-human GH antibodies; incidence of technical complaints; bone age (BA, radiograph of left hand and wrist), centrally assessed according to Greulich and Pyle (23) progression vs CA at week 52; and changes from baseline in clinical safety laboratory parameters, including hematology, biochemistry, hormones (including morning cortisol, thyroid function test), fasting lipids, fasting glucose, fasting insulin, and glycated hemoglobin (HbA_1c_) levels.

### Statistical analysis

The full analysis set (FAS) was used in the analyses of the efficacy end points, except for a sensitivity analysis of the primary end point, for which the per-protocol (PP) analysis set was used. The FAS was to contain all randomly assigned children who received at least 1 dose of treatment. The PP analysis set contained children from the FAS who did not violate any inclusion/exclusion criteria and were administered treatment for at least 22 weeks (for children receiving somapacitan) or 154 days (for children receiving daily GH) during the main trial period. The safety analysis set (SAS) included all randomly assigned children who received at least 1 dose of randomized treatment.

Annualized HV at week 26 and HV at week 52 were analyzed using a mixed model for repeated measurements (MMRM), with treatment, age group, sex, region, and sex by age group interaction term as factors and height at baseline as a covariate, all nested within week as a factor. An unstructured covariance matrix was used to describe the variability for the repeated measurements for a child. From the MMRM, the treatment differences between somapacitan and daily GH treatment arms were estimated with the corresponding 95% CIs.

The secondary efficacy end points of change from baseline in height SDS, HV SDS, IGF-I SDS, and IGFBP-3 SDS were analyzed using a similar MMRM, with baseline assessment as a covariate and treatment, age group, sex, region, and sex by age group interaction term as factors, all nested within week as a factor. For change from baseline in GHD-CIM ObsRO score, this model corresponded to an analysis of covariance model used with treatment, age group, sex, region, and sex by age group interaction as factors and GHD-CIM ObsRO score at baseline as a covariate.

All AEs with onset after the first administration of treatment and up until week 26 (main phase) or 52 (extension period), or 14 days after last dose of trial drug, whichever came first, were included.

### Role of the funding source

The sponsor was involved in the study design, collection, analysis, and interpretation of data, as well as data checking of information provided in the manuscript.

## Results

### Patient characteristics and disposition

**Figure 2.**
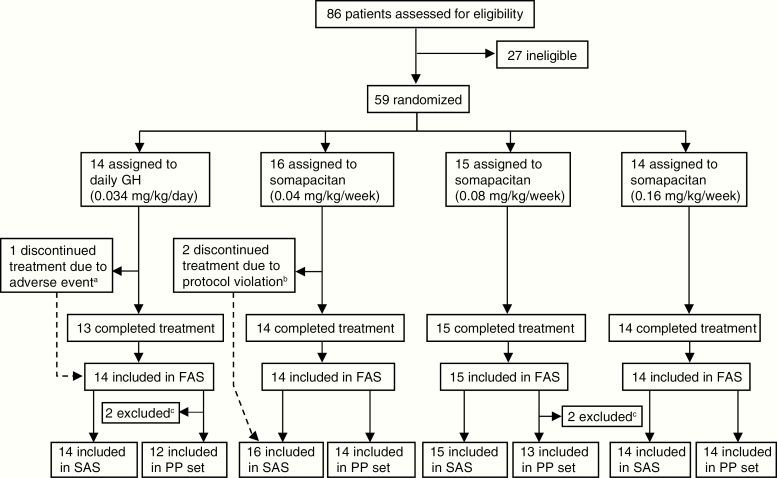
Trial profile. The FAS was to contain all randomly assigned children who received at least 1 dose of randomized treatment. Only in exceptional cases, such as random assignment in error, could children be excluded from the FAS. PP set: children from the FAS who did not violate any inclusion/exclusion criteria and used the randomized treatment for at least 22 weeks (for children receiving somapacitan) or 154 days (for children receiving daily GH) during the main trial period. SAS: all randomly assigned children who received at least 1 dose of randomized treatment. ^a^One child in the daily GH group discontinued because of a nonserious adverse event (mild drug hypersensitivity), which was deemed probably related to treatment. ^b^One child was withdrawn by parents who declined further participation, and the other had a prior history or presence of malignancy and/or intracranial tumor. ^c^One child in the daily GH arm was excluded from the PP set because of not enough exposure (the patient referred to in footnote a). The other 3 children were excluded because of a violation of the height velocity (HV) inclusion criterion. FAS, full analysis set; GH indicates growth hormone; PP, per protocol analysis set; SAS, safety analysis set.

A total of 59 children who were GH treatment naive and prepubertal at screening were randomly assigned. In total, 58 children completed both trial periods and 56 completed the treatment (Fig. 2). Three children discontinued treatment prematurely (see footnote to Fig. 2). Two of these children were excluded from the FAS (and PP analysis set) because they were randomly assigned in error and discontinued treatment after 1.1 and 3.1 weeks of treatment, respectively.

Of the 57 children included in the FAS, 60% were boys. The mean age at baseline was 5.8 to 6.1 years across dose groups (range, 2.8-9.8 years) (Table 1). Adherence was high (mean ≥ 91.8%; median > 99%) for all treatments (Table 2).

**Table 1.** Baseline characteristics

	Somapacitan (0.04 mg/kg/wk)	Somapacitan (0.08 mg/kg/wk)	Somapacitan (0.16 mg/kg/wk)	Daily GH (0.034 mg/kg/d)
FAS, n	14	15	14	14
Male, %	50.0	66.7	57.1	64.3
Age, y	5.8 (1.8)	5.9 (1.8)	6.1 (2.3)	6.0 (2.0)
Weight, kg	14.2 (4.22)	14.0 (3.54)	14.9 (5.23)	15.5 (5.03)
BMI, kg/m^2^	15.3 (1.1)	14.6 (1.1)	15.1 (1.2)	15.6 (1.4)
Height, cm	95.4 (14.0)	97.2 (11.3)	97.7 (16.4)	98.4 (13.8)
Height SDS	−4.1 (1.9)	−3.5 (1.5)	−3.8 (2.0)	−3.4 (1.1)
HV SDS	−2.9 (1.9)	−1.8 (1.7)	−2.9 (1.8)	−3.1 (2.1)
Bone age, y	2.7 (1.3)	3.6 (1.6)	3.7 (2.0)	3.4 (1.8)
GH peak, µg/L	2.9 (2.2)	3.6 (2.1)	4.1 (2.4)	4.0 (2.0)
IGF-I SDS	−2.5 (1.0)	−2.5 (0.8)	−2.0 (1.0)	−2.1 (0.7)
Cause of GHD				
Idiopathic, n	14	14	13	12
Organic, n	–	1	1	2

Data are mean (SD) unless otherwise stated.

Abbreviations: BMI, body mass index; d, day; FAS, full analysis set (all children who were correctly randomly assigned and received at least 1 dose of randomized treatment); GH, growth hormone; GHD, growth hormone deficiency; HV, height velocity; IGF, insulin-like growth factor; n, number of patients; SDS, SD score; wk, week; y, years.

**Table 2.** Treatment adherence over 52 weeks of treatment, according to diary^*a*^ (full analysis set)

	Somapacitan (0.04 mg/kg/wk)	Somapacitan (0.08 mg/kg/wk)	Somapacitan (0.16 mg/kg/wk)	Daily GH (0.034 mg/kg/d)
Mean (SD)	97.5 (4.50)	98.6 (1.66)	96.3 (5.19)	91.8 (23.03)
Median	99.1	100	99.1	99.2
Range	83.6; 100.0	96.2; 100.0	84.9; 100.0	12.3; ^*b*^ 99.7

Abbreviation: GH, growth hormone; wk, week.

^*a*^Number of reported dosings from diary in adherence divided by number of planned dosings multiplied by 100.

^*b*^The lower limit of adherence was due to 1 patient discontinuing treatment after 44 days.

### Efficacy results

#### **Primary end point: annualized HV**.

**Figure 3.**
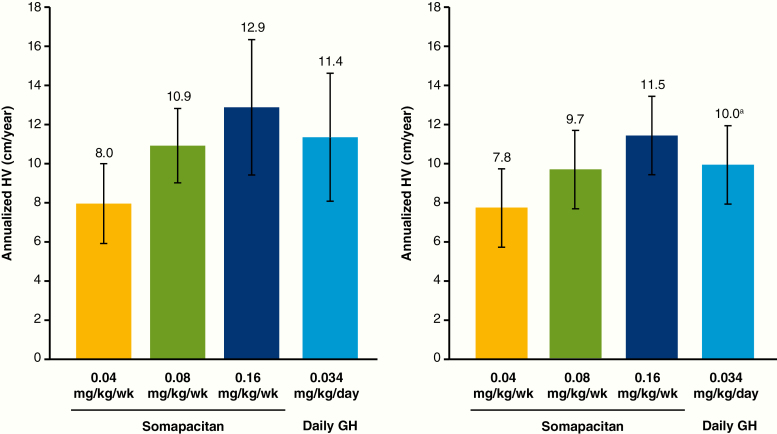
Mean height velocity at week 26 (left) and 52 (right). Data are mean, SD, observed values, full analysis set. Statistical analysis of the end-of-treatment differences is reported in the text. ^a^Value was 9.8 for the full analysis set. The value of 10.0 is from a post hoc-defined analysis that excluded visits after week 4 for the patient in the daily GH group who discontinued treatment prematurely at week 6. GH indicates growth hormone; wk, week.

At week 26, a dose–response relationship with respect to mean observed HV was seen for somapacitan (Fig. 3). Estimated annualized HV (cm/year) was 7.8, 10.9, and 13.1 for somapacitan 0.04, 0.08, and 0.16 mg/kg/week, respectively, and 11.4 for daily GH. At week 26, HV difference was not statistically significant between daily GH and somapacitan 0.16 or 0.08 mg/kg/week; however, HV was significantly greater with daily GH than with somapacitan 0.04 mg/kg/week (Table 3). The sensitivity analysis using the PP analysis set supported the results from the primary analysis.

**Table 3.** Statistical analysis of annualized height velocity

	ETD (95% CI); cm/y	
	Wk 26	Wk 52
Somapacitan 0.16 mg/kg/wk—daily GH	1.7 (–0.2 to 3.6)	1.8 (0.5 to 3.1)
Somapacitan 0.08 mg/kg/wk—daily GH	–0.6 (–2.4 to 1.3)	–0.2 (–1.5 to 1.1)
Somapacitan 0.04 mg/kg/wk—daily GH	–3.7 (–5.6 to –1.8)	–2.4 (–3.7 to –1.1)

Abbreviations: ETD, estimated treatment difference; GH, growth hormone; wk, week; y, year.

At week 52, mean observed HV values were slightly lower than at week 26 in all groups (Fig. 3). Estimated HV was 7.5, 9.7, and 11.7 cm/year, for somapacitan 0.04, 0.08, and 0.16 mg/kg/week, respectively, and 9.9 cm/year for daily GH. HV with somapacitan 0.16 mg/kg/week was significantly greater than with daily GH (Table 3).

#### Other height-related end points.

At week 26, change from baseline in height SDS did not differ between somapacitan 0.16 mg/kg/week or 0.08 mg/kg/week and daily GH, and it was significantly greater with daily GH than with somapacitan 0.04 mg/kg/week (Table 4). At week 52, change from baseline in height SDS was significantly greater with somapacitan 0.16 mg/kg/week compared with daily GH, but did not differ significantly with somapacitan 0.08 mg/kg/week and daily GH. The change was significantly greater with daily GH than with somapacitan 0.04 mg/kg/week (Table 4).

**Table 4.** Statistical analysis of height SDS change from baseline

	ETD (95% CI)	
	Wk 26	Wk 52
Somapacitan 0.16 mg/kg/wk—daily GH	0.16 (–0.06 to 0.38)	0.35 (0.05 to 0.65)
Somapacitan 0.08 mg/kg/wk—daily GH	–0.08 (–0.30 to 0.14)	–0.10 (–0.39 to 0.20)
Somapacitan 0.04 mg/kg/wk—daily GH	–0.44 (–0.66 to –0.22)	–0.58 (–0.88 to –0.28)

Abbreviations: ETD, estimated treatment difference; GH, growth hormone; SDS, SD score; wk, week.

**Figure 4.**
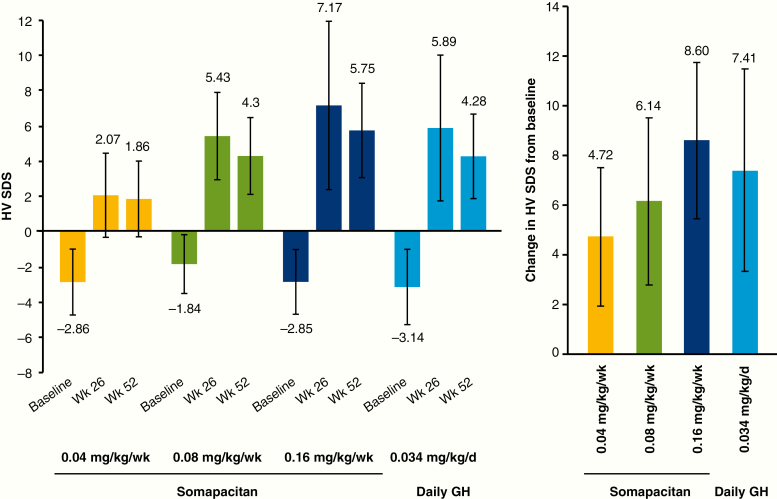
Mean height velocity SDS at baseline, week 26 and week 52 (left), and observed change in height velocity SDS from baseline to week 52 (right). Data are mean (SD), observed values, full analysis set. d, day; GH indicates growth hormone; HV SDS, height velocity SD score; wk, week.

**Figure 5.**
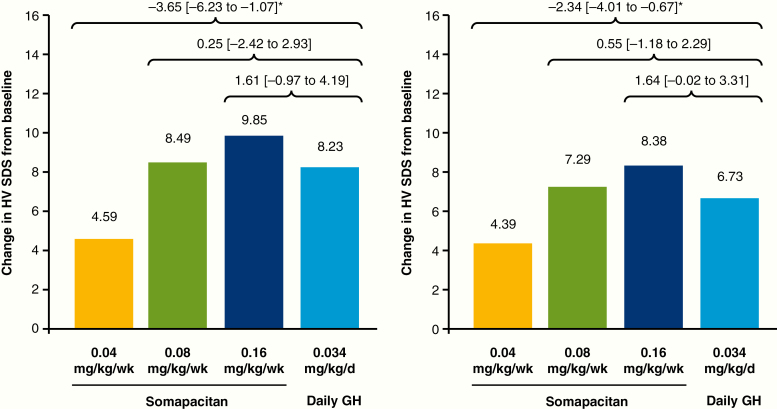
Mean height velocity SDS: change from baseline to week 26 (left) and week 52 (right). Data are estimated mean values. Numbers above brackets are estimated treatment differences (weekly somapacitan–daily GH) and 95% CI. *Denotes statistical significance. d, day; GH indicates growth hormone; HV SDS, height velocity SD score; wk, week.

The results for HV SDS were similar to those for the primary end point: HV SDS increased with somapacitan dose, and values at week 52 were slightly lower than those at week 26 (Fig. 4). The observed increase in HV SDS with somapacitan increased dose-dependently (Fig. 4). At week 26, the change in HV SDS from baseline did not differ between somapacitan 0.16 mg/kg/week or 0.08 mg/kg/week and daily GH (Fig. 5). Change in HV SDS was greater with daily GH than with somapacitan 0.04 mg/kg/week. Similar results were obtained at week 52.

#### Observed insulin-like growth factor-I SD score.

At week 26, the mean (SD) IGF-I SDS observed at the peak of the profile (days 1-4 after dose) increased from a mean baseline value below or at −2 (Table 1) to mean (SD) values of −1.46 (1.21), −0.60 (1.25), and +0.97 (1.49), for somapacitan 0.04, 0.08, and 0.16 mg/kg/week, respectively. The observed IGF-I SDS at week 26 for daily GH was –0.22 (0.81). At week 52, IGF-I SDS values observed at the peak of the profile were −1.41 (1.19), −0.48 (1.08), and +1.25 (1.72) for somapacitan 0.04, 0.08, and 0.16 mg/kg/week, respectively. The observed IGF-I SDS at week 52 for daily GH was –0.40 (1.50). The numerical decrease in mean IGF-I SDS for daily GH between weeks 26 and 52 was likely due to 1 patient who was no longer receiving treatment at week 52.

The estimated treatment differences (ETDs) in change from baseline to week 26 and 52 were significantly greater for somapacitan 0.16 mg/kg/week, similar for somapacitan 0.08 mg/kg/week, and smaller for somapacitan 0.04 mg/week, compared with daily GH (Table 5).

**Table 5.** Statistical analysis of change from baseline in IGF-I SDS

	ETD (95% CI)	
	Wk 26	Wk 52
Somapacitan 0.16 mg/kg/wk—daily GH	1.13 (0.38 to 1.89)	1.56 (0.66 to 2.46)
Somapacitan 0.08 mg/kg/wk—daily GH	–0.10 (–0.88 to 0.69)	–0.05 (–0.86 to 0.97)
Somapacitan 0.04 mg/kg/wk—daily GH	−0.99 [−1.76 to −0.22)	−0.85 (−1.78 to −0.08)

Week 26 and week 52 samples for determining IGF-I levels were taken at peak levels of somapacitan (days 1-4 after dosing). Observed IGF-I SDS values corresponding to peak and trough levels of somapacitan, and derived average values, are shown in Fig. 8.

Abbreviations: ETD, estimated treatment difference; GH, growth hormone; IGF-I, insulin-like growth factor-I; SDS, SD score; wk, week.

#### Model-derived insulin-like growth factor-I SD score.

**Figure 6.**
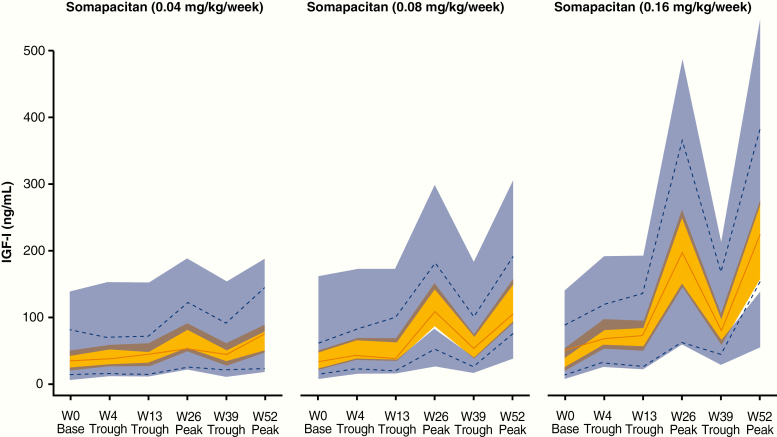
Visual predictive check for the population PK/PD model for somapacitan by dose level. Full red lines indicate observed median insulin-like growth factor-I (IGF-I) and dotted blue lines indicate the 5th to 95th percentile by visit. Yellow and blue areas indicate the model predicted 95% CI around the median and 5th to 95th percentile, respectively. Baseline samples (W0) were taken before first dose, trough samples (W4, W13, and W39) were taken 7 days after dose, and peak samples (W26 and W52) were taken 1 to 4 days after dose. PK/PD indicates pharmacokinetic/pharmacodynamic; W, week.

**Figure 7.**
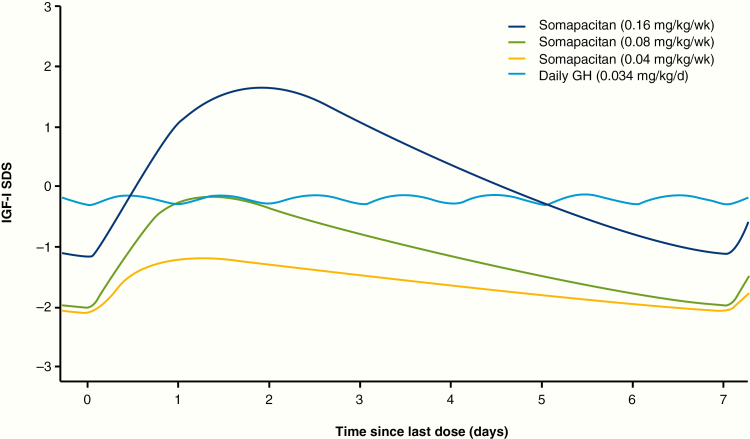
Modeled IGF-I SDS fluctuations over the week. Insulin-like growth factor-I (IGF-I) SDS profiles for somapacitan were derived by population PK/PD modeling from the REAL 3 trial. Daily GH IGF-I SDS profiles are shown for reference and were derived by population PK/PD modeling of phase 1 data (daily GH model not published). d, day; GH indicates growth hormone; PK/PD, pharmacokinetic/pharmacodynamic; SDS, SD score; wk, week.

**Figure 8.**
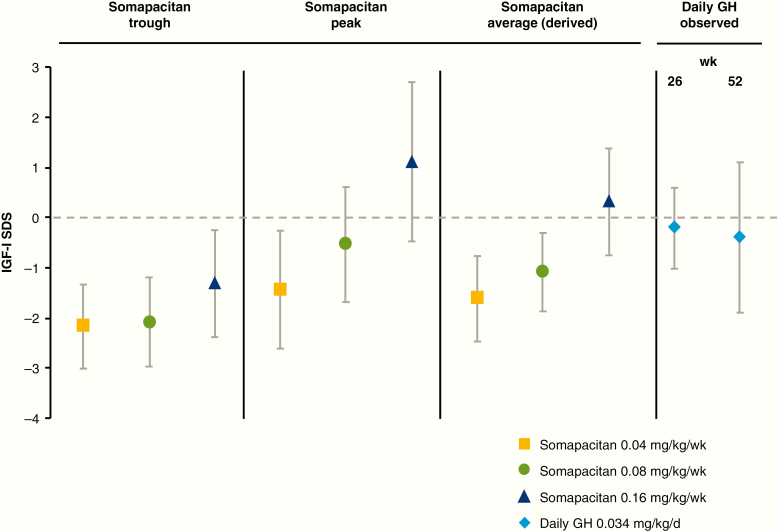
Mean IGF-I SDS: observed and derived values. Somapacitan trough values are an average from values obtained 7 days after dose at weeks 4, 13, and 39. Somapacitan peak values are an average from values obtained 1 to 4 days after dose at weeks 26 and 52. Somapacitan average values were derived by population PK/PD modeling. Daily GH IGF-I levels from week 26 and 52 are shown for reference. Points and bars are means with SD. d, day; GH indicates growth hormone; PK/PD, pharmacokinetic/pharmacodynamic; SDS, SD score; wk, week.

A total of 212 PK samples (excluding baseline) and 252 IGF-I samples (including baseline) were available from 43 patients treated with somapacitan. Four PK samples (1.9% of total) and 7 IGF-I samples (2.8% of total) were excluded because of missing or ambiguous dose records. The population PK/PD model demonstrated good predictive properties for baseline, peak, and trough samples at all visits for all dose levels based on visual predictive checks (Fig. 6). Thus, the population PK/PD model was judged adequate to derive individual IGF-I profiles and IGF-I average levels during a dosing interval and to derive individual IGF-I average levels based on the available PK and IGF-I data. IGF-I averages were derived on an ng/mL scale and converted to SDS (18). The predicted IGF-I profile following somapacitan peaked between day 1 and 2 after dosing, with a fluctuation over the week that was greater than that of daily GH (Fig. 7). The mean (SD) of the model-derived IGF-I average SDS values over the week for somapacitan 0.04, 0.08, and 0.16 mg/kg/week were −1.62 (0.86), −1.09 (0.78), and 0.31 (1.06), respectively (Fig. 8). The observed IGF-I levels in trough samples (weeks 4, 13, and 39) and peak samples (weeks 26 and 52) for somapacitan and for daily GH (weeks 26 and 52) are also shown in Fig. 8. The average IGF-I SDS obtained with somapacitan 0.16 mg/kg/week was the closest to that of daily GH (Fig. 8).

Five patients in the somapacitan 0.16 mg/kg/weekly group had IGF-I SDS values greater than 2: 1 at week 26 only, 2 at week 52 only, 1 at weeks 26 and 52, and 1 at weeks 26, 39, and 52.

#### Insulin-like binding protein 3 SD score.

At week 26, mean (SD) IGFBP-3 SDS values observed on days 1 to 4 after somapacitan dose were –1.29 (1.05), –0.83 (1.37), and –0.11 (1.07) for somapacitan 0.04, 0.08, and 0.16 mg/kg/week, respectively. At week 52, the corresponding values were –1.22 (1.21), –0.80 (0.83), and 0.27 (0.97), respectively. Observed mean IGFBP-3 SDS values for daily GH 0.034 mg/kg/day were –0.15 (0.82) at week 26 and –0.74 (1.56) at week 52.

#### Patient-reported outcomes.

**Figure 9.**
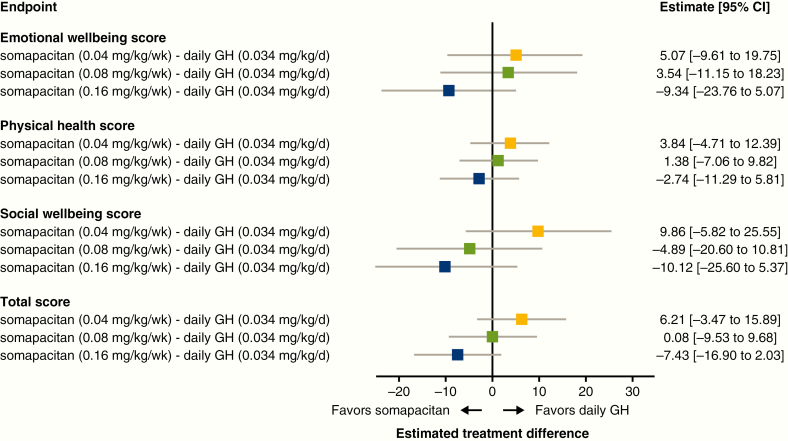
Change from baseline in Growth Hormone Deficiency—Child Impact Measure observer (GHD-CIM ObsRO) scores after 52 weeks of treatment (full analysis set). Patient and clinician ratings of severity of disease suggest a minimal important difference (MID) of 5 points for the total score, 7 for emotional well-being, 5 for physical health, and 5 for the social well-being domains, with a lower score representing an improvement. d, day; GH indicates growth hormone; wk, week.

The change from baseline in the GHD-CIM ObsRO was compared between daily GH and each dose of somapacitan. Eleven of 57 children analyzed were younger than 4 years. With somapacitan 0.16 mg/kg/week, an improvement over daily GH was observed across all domains as well as the total score, although treatment differences vs daily GH were not statistically significant (Fig. 9). ETDs between somapacitan 0.16 mg/kg/week and daily GH at week 52 exceeded the MID in favor of somapacitan for the emotional well-being and social well-being domains and the total score.

### Safety results

The results are based on the SAS including all children exposed (59 children). Somapacitan was well tolerated at all 3 doses during both the main and extension periods, with no new clinically significant safety or local tolerability issues identified.

**Figure 10.**
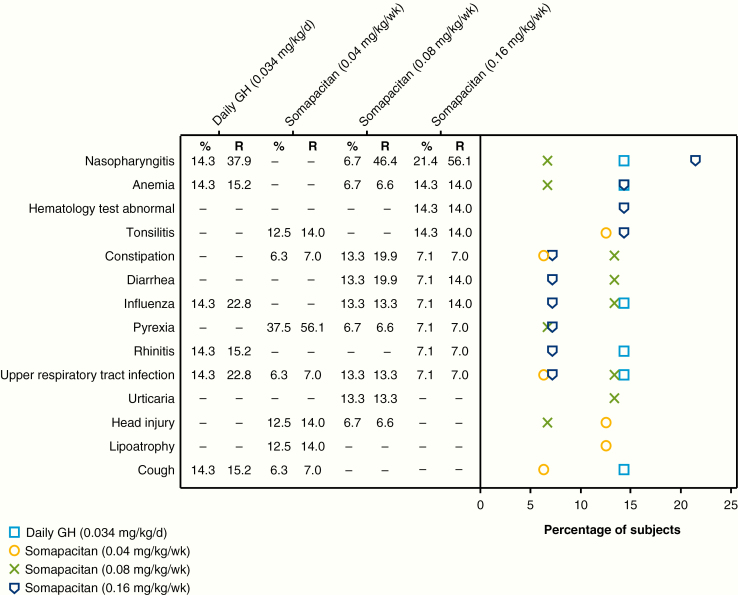
Most common adverse events, occurring in 10% or more of patients in any patient group (safety analysis set). % indicates percentage of patients; d, day; GH, growth hormone; R, event rate per 100 patient-years at risk; wk, week.

Overall, AE rates per 100 patient-years were similar between the somapacitan 0.16 mg/kg/week group (364.8) and the daily GH group (364.1), with lower rates in the somapacitan 0.04 mg/kg/week (196.3) and 0.08 mg/kg/week (304.9) groups (Table 6). The most common AEs are shown in Fig. 10 and were mainly events commonly associated with GH therapy.

**Table 6.** Adverse events in main trial and extension (safety analysis set)

	Somapacitan (0.04 mg/kg/wk)				Somapacitan (0.08 mg/kg/wk)				Somapacitan (0.16 mg/kg/wk)				Daily GH (0.034 mg/kg/d)			
	N	(%)	E	R	N	(%)	E	R	N	(%)	E	R	N	(%)	E	R
Patients exposed	16				15				14				14			
**All events**	**10**	**(62.5)**	**28**	**196.3**	**11**	**(73.3)**	**46**	**304.9**	**13**	**(92.9)**	**52**	**364.8**	**14**	**(100.0)**	**48**	**364.1**
Mild events	7	(43.8)	15	105.2	11	(73.3)	44	291.6	11	(78.6)	42	294.6	14	(100.0)	44	333.8
Moderate events	4	(25.0)	11	77.1	1	(6.7)	2	13.3	6	(42.9)	10	70.1	3	(21.4)	4	30.3
Severe events	2	(12.5)	2	14.0	0				0				0			
Probably related	2	(12.5)	2	14.0	0				1	(7.1)	2	14.0	1	(7.1)	1	7.6
Possibly related	0				1	(6.7)	2	13.3	1	(7.1)	1	7.0	0			
Unlikely related	10	(62.5)	26	182.3	11	(73.3)	44	291.6	13	(92.9)	49	343.7	14	(100.0)	47	356.5

Only adverse events with an onset after the first administration of trial product and up until week 52 or 14 days after last trial drug administration, whichever comes first, are included. Values in bold are “All events”. All others are sub-categories.

Abbreviations: %, percentage of patients; d, day; E, number of events; GH, growth hormone; N, number of patients; R, event rate per 100 patient-years at risk; wk, week.

There were 7 serious AEs in 3 children: 1 child in the somapacitan 0.08 mg/kg/week group reported multiple pituitary deficiency (mild, unlikely related to treatment) and acute gastroenteritis (moderate, unlikely related); the somapacitan dose was unchanged. This child had a history of pituitary and pituitary stalk hypoplasia and ectopic neurohypophysis. One child in the somapacitan 0.16 mg/kg/week group reported generalized edema (moderate, probably related), vomiting (moderate, probably related), and tonsillitis (moderate, unlikely related); administration of somapacitan was interrupted for the first 2 events, which occurred on successive days, and the child received ceftriaxone for infection. One child in the daily GH group reported bronchitis/respiratory syncytial virus infection (moderate, unlikely related) and nephrotic syndrome (moderate, unlikely related); daily GH was withdrawn as a result of the latter serious AE.

### Injection-site reactions

Two children treated with somapacitan 0.08 mg/kg/week had mild urticaria in week 2 and recovered after 6 to 13 days. One child treated with somapacitan 0.04 mg/kg/week experienced 2 injection-site reactions: injection-site hematoma in week 12, with recovery after 5 days, and lipoatrophy from week 23 to 53.

### Glucose metabolism

There were no clinically relevant changes from baseline to week 26 or week 52 in mean HbA_1c_, fasting glucose, or insulin for any of the treatment groups. Some variation in mean fasting insulin was observed at baseline, week 26, and week 52. At baseline, mean fasting insulin levels for the somapacitan groups were 10.6, 8.8, 21.9, and 19.1 pmol/L for somapacitan 0.04, 0.08, and 0.16 mg/kg/week and daily GH, respectively. Values increased in all groups at week 26 (data not shown) and had stabilized or decreased at week 52, when the values were 26.0, 30.4, 56.9, and 40.6 pmol/L for somapacitan 0.04, 0.08, and 0.16 mg/kg/week and daily GH, respectively.

### Other safety assessments

**Figure 11.**
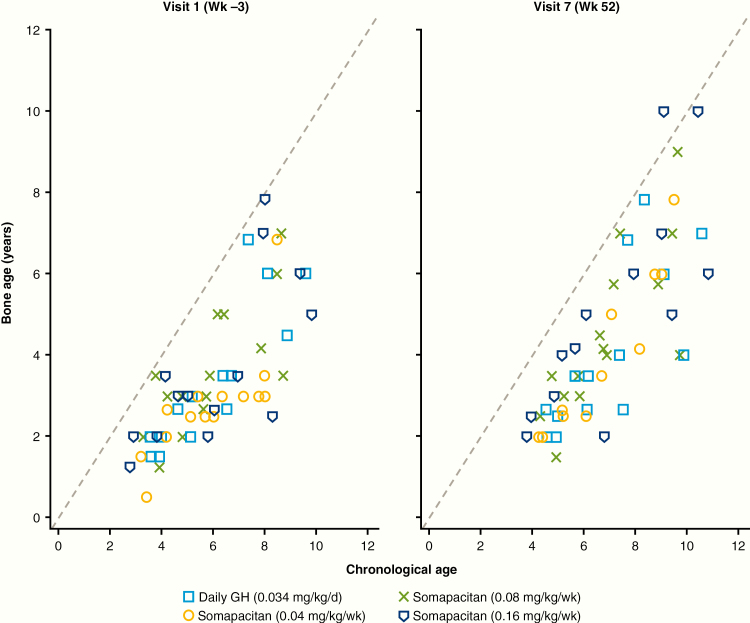
Bone age progression vs chronological age, by visit. d, day; GH indicates growth hormone; wk, week.

No marked change in BA progression vs CA among the treatment groups was noted (Fig. 11). BA increased slightly in all groups, with a change from baseline in BA/CA ratio of 0.09, 0.03, 0.09, and 0.02 for somapacitan 0.04 mg/kg/week, 0.08 mg/kg/week, 0.16 mg/kg/week, and daily GH, respectively. No changes in skeletal proportions were reported.

No clinically relevant findings were observed for other safety assessments (physical examination, vital signs, laboratory assessments). Mean alkaline phosphatase increased from baseline to week 52 in all groups; the increases with somapacitan were similar to those observed with daily GH. No neutralizing anti-hGH or antisomapacitan antibodies were detected. One child in the daily GH group had persistent nonneutralizing anti-hGH antibodies of low titer. Six patients in the somapacitan groups had 1 single positive transient measurement of low-titer, nonneutralizing, antisomapacitan antibodies (2 patients each in the 0.04, 0.08, and 0.16 mg/kg/week groups, respectively). Antibodies did not appear to affect PK or PD profiles or annualized HV.

## Discussion

The doses of somapacitan used in this trial were selected with the aim of identifying the dose that best matched that of daily GH with respect to HV end points, IGF-I levels, and overall safety. In children with GHD, the safety and tolerability of once-weekly somapacitan 0.04, 0.08, and 0.16 mg/kg/week were similar to those of daily GH 0.034 mg/kg/day. Efficacy was similar for the somapacitan 0.08 and 0.16 mg/kg/week doses compared with daily GH, with the 0.16 mg/kg/week dose providing the closest efficacy match based on HV, change in HV SDS, and average IGF-I SDS levels at week 26. The results of the extension phase supported the findings from the main phase, and at week 52, HV was statistically significantly greater with somapacitan 0.16 mg/kg/week than with daily GH. HV values at 12 months were in line with those reported for other studies of long-acting GHs (24, 25). Weekly IGF-I average levels for the somapacitan 0.16 mg/kg dose provided the best match for the average IGF-I levels observed for daily GH.

With any long-acting GH formulation, the fluctuation in IGF-I levels will differ significantly compared to IGF-I levels observed with daily GH because of different modes of action and PK and PD profile (4). For somapacitan, IGF-I was sampled to characterize the profile over the week with samples around the peak (days 1-4 after injection) and at trough (day 7 after injection). PK/PD modeling showed IGF-I levels increasing to a maximum at days 1 to 2 and then tapering downward toward trough levels. Thus, with somapacitan, observed IGF-I SDS peak values were above and trough values were below the IGF-I levels observed with daily GH, whereas the model-derived average weekly IGF-I SDS was comparable with the IGF-I levels observed with daily GH.

IGF-I from samples collected on days 1 to 2 are expected to be above the average. Although the population mean peak level following 0.16 mg/kg/week somapacitan was predicted to be below 2 SDS, some patients treated to reach normal average IGF-I levels are expected to be above 2 SDS for a transient period (eg, days 1-2) during the weekly interval. This was also observed in the peak (days 1-4) samples. However, the trough levels were correspondingly lower, with a population mean around –1 SDS at trough, resulting in a weekly IGF-I average level similar to that of daily GH.

Somapacitan was well tolerated at all dose levels investigated. AEs were mild to moderate; most were evaluated as unlikely related to treatment by the investigator. Tolerability was consistent with that of daily GH. Thus, based on HV, change in HV SDS, overall IGF-I SDS values, and tolerability, the somapacitan dose for the phase 3 trial in children with GHD will be 0.16 mg/kg/week.

Numerous attempts have been made to develop long-acting GH formulations, but most have encountered setbacks, frequently relating to injection-site reactions. In contrast to 2 previous phase 3 studies of long-acting GHs, very few injection-site reactions were reported with somapacitan. Reiter et al reported a high incidence of administration-site AEs with Nutropin Depot (26), which was withdrawn from use in children with GHD in 2004. Khadilkar and colleagues reported a higher incidence of injection-site reactions with LB03002 than with daily GH (24).

The goal of developing long-acting formulations is to lessen the burden of daily injections and thus potentially improve treatment adherence. Adherence was high in all treatment groups (mean 97.5%, 98.6%, and 96.3% in the somapacitan 0.04, 0.08, and 0.16 mg/kg/week groups, respectively, and 91.8% in the daily GH group). High levels of adherence are expected in a controlled clinical trial but are not necessarily reflected in real life (27).

The reduction in the number of injections required with weekly vs daily GH treatment (52 vs more than 350 per year) is expected to reduce the treatment burden on patients and their caregivers, decreasing disruption to daily life. When comparing treatments using the GHD-CIM ObsRO, the ETD between somapacitan 0.16 mg/kg/week and daily GH at week 52 exceeded the MID for the total score, and for the emotional well-being and social well-being domains, suggesting clinically relevant improvement for these parameters in favor of somapacitan.

This trial had some limitations. For example, blinding was not possible for the once-weekly somapacitan doses vs daily GH. Treatment group sizes were small but appropriate for a dose-finding trial in a rare disease such as GHD. The strengths of this study were that the 3 somapacitan doses were blinded, that the 26-week efficacy and safety results were confirmed by the 52-week results, and that it is to our knowledge the first trial of a long-acting GH to report disease-specific patient-reported outcome assessments in children with GHD.

The present data provide strong support that once-weekly somapacitan is efficacious in children with GHD with a favorable safety profile. Efficacy of the 0.16 mg/kg/week doses was similar to that of daily GH judged by several measures, and at week 52, HV was statistically significantly greater with somapacitan 0.16 mg/kg/week compared with daily GH. Scores of emotional and social well-being were improved with somapacitan 0.16 mg/kg/week compared with daily GH. Safety and tolerability of all doses of once-weekly somapacitan were similar to those of daily GH. Somapacitan was well tolerated at all doses investigated, with no new safety or local tolerability issues (including injection-site reactions) identified. A global phase 3 trial (REAL 4; NCT03811535) has been initiated to further investigate the efficacy and safety of once-weekly somapacitan in a large cohort of children with GHD.

## Abbreviations

ETD: estimated treatment difference
GHD: growth hormone deficiency
HV: height velocity
IGF-I: insulin-like growth factor-I

## Additional Information

***Disclosure Summary:*** L.S. has received consultancy fees from Ascendis, Hexal, Novo Nordisk, Merck, Pfizer, and Sandoz. T.B. has received grants and consultancy fees from Novo Nordisk; personal fees from Eli Lilly; and grants from Sandoz, National Institute of Diabetes and Digestive and Kidney Diseases–National Institutes of Health, the Slovenian National Research Agency, and the Helmsley Foundation. M.B. has received consultancy fees from Novo Nordisk. M.H.R. is an employee of and stockholder in Novo Nordisk. R.H. has received grants and consultancy fees from Novo Nordisk Pharma Ltd, grants from Sandoz, and consultancy fees from Pfizer and Genxine. R.V.J. is an employee of and stockholder in Novo Nordisk. P.S. is a clinical investigator for Ascendis, OPKO, and Novo Nordisk.

***Data Availability:*** The study protocol and redacted clinical study reports are available according to Novo Nordisk data-sharing commitments. Individual participant data have been shared with the study investigators in a pseudonymized format. The data will be available as redacted study reports permanently after research completion and approval of product use both in the European Union and the United States with no end date. Data will be shared with bona fide researchers submitting a research proposal requesting access to data, for analyses in line with the aims of the study protocol and approved by the independent review board according to the independent review board charter (see novonordisk-trials.com). Request proposal forms and the access criteria can be found at novonordisk-trials.com. The data will be made available on a specialized SAS data platform.

## References

[1] *Sävendahl L, Battelino T, Brod M, et al. Animated summary* https://nn-product.videomarketingplatform.co/secret/61057287/48b6ad3a8535c52bc0b2eb60c471c319

[2] Grimberg A, DiVall SA, Polychronakos C, et al; Drug and Therapeutics Committee and Ethics Committee of the Pediatric Endocrine Society Guidelines for growth hormone and insulin-like growth factor-I treatment in children and adolescents: growth hormone deficiency, idiopathic short stature, and primary insulin-like growth factor-I deficiency. Horm Res Paediatr. 2016;86(6):361–397. 27884013 10.1159/000452150

[3] Acerini CL, Segal D, Criseno S, et al. Shared decision-making in growth hormone therapy—implications for patient care. Front Endocrinol (Lausanne). 2018;9:688. 30524377 10.3389/fendo.2018.00688 PMC6262035

[4] Yuen KCJ, Miller BS, Biller BMK The current state of long-acting growth hormone preparations for growth hormone therapy. Curr Opin Endocrinol Diabetes Obes. 2018;25(4):267–273. 29746309 10.1097/MED.0000000000000416

[5] Kapoor RR, Burke SA, Sparrow SE, et al. Monitoring of concordance in growth hormone therapy. Arch Dis Child. 2008;93(2):147–148. 17768149 10.1136/adc.2006.114249

[6] Cutfield WS, Derraik JG, Gunn AJ, et al. Non-compliance with growth hormone treatment in children is common and impairs linear growth. PloS One. 2011;6(1):e16223. 21305004 10.1371/journal.pone.0016223 PMC3031542

[7] Battelino T, Rasmussen MH, De Schepper J, Zuckerman-Levin N, Gucev Z, Sävendahl L; NN8640-4042 Study Group Somapacitan, a once-weekly reversible albumin-binding GH derivative, in children with GH deficiency: a randomized dose-escalation trial. Clin Endocrinol. 2017;87(4):350–358. 10.1111/cen.13409 28656605

[8] Havelund S, Plum A, Ribel U, et al. The mechanism of protraction of insulin detemir, a long-acting, acylated analog of human insulin. Pharm Res. 2004;21(8):1498–1504. 15359587 10.1023/b:pham.0000036926.54824.37

[9] Deacon CF Potential of liraglutide in the treatment of patients with type 2 diabetes. Vasc Health Risk Manag. 2009;5(1):199–211. 19436648 10.2147/vhrm.s4039 PMC2672437

[10] Lau J, Bloch P, Schäffer L, et al. Discovery of the once-weekly glucagon-like peptide-1 (GLP-1) analogue semaglutide. J Med Chem. 2015;58(18):7370–7380. 26308095 10.1021/acs.jmedchem.5b00726

[11] Rasmussen MH, Olsen MW, Alifrangis L, Klim S, Suntum M A reversible albumin-binding growth hormone derivative is well tolerated and possesses a potential once-weekly treatment profile. J Clin Endocrinol Metab. 2014;99(10):E1819–E1829. 25013997 10.1210/jc.2014-1702

[12] Rasmussen MH, Janukonyté J, Klose M, et al. Reversible albumin-binding GH possesses a potential once-weekly treatment profile in adult growth hormone deficiency. J Clin Endocrinol Metab. 2016;101(3):988–998. 26727076 10.1210/jc.2015-1991 PMC4803179

[13] Johannsson G, Feldt-Rasmussen U, Håkonsson IH, et al; REAL 2 Study Group Safety and convenience of once-weekly somapacitan in adult GH deficiency: a 26-week randomized, controlled trial. Eur J Endocrinol. 2018;178(5):491–499. 29500310 10.1530/EJE-17-1073 PMC5920019

[14] Juul RV, Rasmussen MH, Agersø H, Overgaard RV Pharmacokinetics and pharmacodynamics of once-weekly somapacitan in children and adults: supporting dosing rationales with a model-based analysis of three phase I trials. Clin Pharmacokinet. 2019;58(1):63–75. 29671202 10.1007/s40262-018-0662-5 PMC6325982

[15] Tanner JM, Whitehouse RH Clinical longitudinal standards for height, weight, height velocity, weight velocity, and stages of puberty. Arch Dis Child. 1976;51(3):170–179. 952550 10.1136/adc.51.3.170 PMC1545912

[16] Kuczmarski RJ, Ogden CL, Grummer-Strawn LM, et al CDC growth charts: United States. Adv Data. 2000;(314):1–27. 11183293

[17] Prader A, Largo RH, Molinari L, Issler C Physical growth of Swiss children from birth to 20 years of age. First Zurich longitudinal study of growth and development. Helv Paediatr Acta Suppl. 1989;52:1–125. 2737921

[18] Bidlingmaier M, Friedrich N, Emeny RT, et al. Reference intervals for insulin-like growth factor-1 (IGF-I) from birth to senescence: results from a multicenter study using a new automated chemiluminescence IGF-I immunoassay conforming to recent international recommendations. J Clin Endocrinol Metab. 2014;99(5):1712–1721. 24606072 10.1210/jc.2013-3059

[19] Friedrich N, Wolthers OD, Arafat AM, et al. Age- and sex-specific reference intervals across life span for insulin-like growth factor binding protein 3 (IGFBP-3) and the IGF-I to IGFBP-3 ratio measured by new automated chemiluminescence assays. J Clin Endocrinol Metab. 2014;99(5):1675–1686. 24483154 10.1210/jc.2013-3060

[20] Lindbom L, Pihlgren P, Jonsson EN, Jonsson N PsN-Toolkit—a collection of computer intensive statistical methods for non-linear mixed effect modeling using NONMEM. Comput Methods Programs Biomed. 2005;79(3):241–257. 16023764 10.1016/j.cmpb.2005.04.005

[21] Vienna, Austria: R Foundation for Statistical Computing R Version 3. R website. https://www.r-project.org/. Accessed September 2019.

[22] US Department of Health and Human Services. Food and Drug Administration. Guidance for industry: patient-reported outcome measures: use in medical product development to support labeling claims FDA website. 2009 https://www.fda.gov/media/77832/download. Accessed June 23, 2019.

[23] Greulich WW, Pyle SJ. Radioagraphic Atlas of Skeletal Development of the Hand and Wrist. Palo Alto, CA: Stanford University Press; 1973.

[24] Khadilkar V, Radjuk KA, Bolshova E, et al. 24-month use of once-weekly GH, LB03002, in prepubertal children with GH deficiency. J Clin Endocrinol Metab. 2014;99(1):126–132. 24170106 10.1210/jc.2013-2502

[25] Zelinska N, Iotova V, Skorodok J, et al. Long-acting c-terminal peptide-modified hGH (MOD-4023): results of a safety and dose-finding study in GHD children. J Clin Endocrinol Metab. 2017;102(5):1578–1587. 28323965 10.1210/jc.2016-3547

[26] Reiter EO, Attie KM, Moshang T Jr, et al; Genentech, Inc-Alkermes, Inc Collaborative Study Group A multicenter study of the efficacy and safety of sustained release GH in the treatment of naive pediatric patients with GH deficiency. J Clin Endocrinol Metab. 2001;86(10):4700–4706. 11600528 10.1210/jcem.86.10.7932

[27] Haverkamp F, Johansson L, Dumas H, et al. Observations of nonadherence to recombinant human growth hormone therapy in clinical practice. Clin Ther. 2008;30(2):307–316. 18343269 10.1016/j.clinthera.2008.02.017

