# Corrigendum to: “Once-Weekly Somapacitan vs Daily GH in Children With GH Deficiency: Results From a Randomized Phase 2 Trial”

**DOI:** 10.1210/clinem/dgaa614

**Published:** 2020-09-30

In the above-named article by Sävendahl L, Battelino T, Brod M, Højby Rasmussen M, Horikawa R, Vestergaard Juul R, Saenger P, on behalf of the REAL 3 Study Group (*J Clin Endocrinol Metab*. 2020;105(4):1847–1861; doi: 10.1210/clinem/dgz310) the following omission occurred in the published paper: “The name of Tülay Güran was omitted from the list of Turkish investigators acknowledged as members of the REAL 3 Study Group.” The correct group membership section should read as follows:

“*Members of the REAL 3 Study Group:

Austria: Dieter Furthner, Bettina Piringer, Lorenz Auer-Hackenberg, Klaus Schmitt, Marlene Reitmayr

Brazil: Marcello Delano Bronstein, Francisco Samuel Magalhães Lima

Germany: Martin Wabitsch, Carsten Posovszky, Volker Böttcher, Alexander Mann

Israel: Eli Hershkovitz, Alon Haim, Neta Lowenthal, Orit Hamiel, Sharon Sheinvald Levin, Kineret Mazor-Aronovitch, Michal Ben-Ami, Yael Levy Shraga, Dalit Modan, Noah Gruber, Moshe Phillip, Yael Lebenthal, Ariel Tenenbaum, Alon Eliakim, Nitzan Dror, Ruby Haviv, Nehama Zuckerman-Levin, Naim Shehadeh, Liav Givon, Ameer Elemy, Miriam Marji, Vardit Gepstein

India: Praveen V.P., Aswin P, Nithiya Abraham, Rajesh Khadgawat, Yashdeep Gupta, Vaman Khadilkar, Anuradha Khadilkar, Sagar Lad

Japan: Reiko Horikawa, Yasuhiro Naiki, Yasuko Ogiwara, Yuta Chiba, Yusuke Fujisawa, Yumiko Terada, Tomoko Yoshida, Kenichi Kinjo, Atsushi Tsukamura, Shinobu Ida, Yuri Etani, Yasuko Shoji, Masanobu Kawai, Hisakazu Nakajima, Jun Mori, Shota Fukuhara, Keiichi Shigehara, Hidechika Morimoto, Yusuke Tsuma, Yasuhiro Kawabe, Takeshi Ota, Kenichi Kashimada, Ryuichi Nakagawa, Atsumi Tsuji, Risa Nomura, Kei Takasawa, Takeru Yamauchi, Kanako Ishii, Naoko Toda, Kazuhiro Ohkubo, Tohru Yorifuji, Yuki Hosokawa, Rie Kawakita, Yukiko Hashimoto, Azumi Sakakibara, Shinji Higuchi, Shun Soneda, Kenichiro Ogushi, Shuichi Yatsuga, Yasutoshi Koga, Takako Matsumoto, Miyuki Kitamura

Sweden: Lars Sävendahl, Ricard Nergårdh

Slovenia: Tadej Battelino, Mojca Zerjav Tansek

Turkey: Serap Turan, Abdullah Bereket, Zeynep Atay, Azad Akbarzade, Tülay Güran

Ukraine: Olena Bolshova, Mykola Tronko, Olga Vyshnevskaya, Natalia Sprynchuk, Iryna Lukashuk, Natalia Muz, Tatyana Marchenko, Nataliya Chorna, Marіana Konovalova, Liliya Zelinska

USA: Lawrence Silverman, Barbara Cerame, Sunita Cheruvu, Daisy Chin, Laurie Ebner-Lyon, Marie Fox, Marianna Nicolette-Gentile, Kristin Sabanosh, Harold Starkman, Ian Marshall, Mariam Gangat, Sadana Balachandar, Philippe Backeljauw, Andrew Dauber, Leah Tyzinski, Paul H. Saenger, Luis Zamora Siliezar, Jacqueline P. Velasco, Judith L. Ross, Martha Bardsley, Karen Kowal, Gad B. Kletter, Britney G. Frazier, Kathryn Garrison”

doi: 10.1210/clinem/dgz310

